# Awakening Neuropsychiatric Research Into the Stria Medullaris: Development of a Diffusion-Weighted Imaging Tractography Protocol of This Key Limbic Structure

**DOI:** 10.3389/fnana.2018.00039

**Published:** 2018-05-08

**Authors:** Darren W. Roddy, Elena Roman, Shane Rooney, Sinaoife Andrews, Chloe Farrell, Kelly Doolin, Kirk J. Levins, Leonardo Tozzi, Paul Tierney, Denis Barry, Thomas Frodl, Veronica O’Keane, Erik O’Hanlon

**Affiliations:** ^1^REDEEM Group, Department of Psychiatry, Trinity College Dublin, Dublin, Ireland; ^2^Department of Anaesthesia, Intensive Care and Pain Medicine, St. Vincent’s Hospital, Dublin, Ireland; ^3^Department of Psychiatry and Psychotherapy, University of Magdeburg, Magdeburg, Germany; ^4^Department of Anatomy, Trinity College Dublin, Dublin, Ireland

**Keywords:** stria medullaris, DTI, tractography, CSD, habenula, depression, deep brain stimulation, limbic system

## Abstract

The Stria medullaris (SM) Thalami is a discrete white matter tract that directly connects frontolimbic areas to the habenula, allowing the forebrain to influence midbrain monoaminergic output. Habenular dysfunction has been shown in various neuropsychiatric conditions. However, there exists a paucity of research into the habenula’s principal afferent tract, the SM. Diffusion-weighted tractography may provide insights into the properties of the SM *in vivo*, opening up investigation of this tract in conditions of monoamine dysregulation such as depression, schizophrenia, addiction and pain. We present a reliable method for reconstructing the SM using diffusion-weighted imaging, and examine the effects of age and gender on tract diffusion metrics. We also investigate reproducibility of the method through inter-rater comparisons. In consultation with neuroanatomists, a Boolean logic gate protocol was developed for use in *ExploreDTI* to extract the SM from constrained spherical deconvolution based whole brain tractography. Particular emphasis was placed on the reproducibility of the tract, attention to crossing white matter tract proximity and anatomical consistency of anterior and posterior boundaries. The anterior commissure, pineal gland and mid point of the thalamus were defined as anatomical fixed points used for reconstruction. Fifty subjects were scanned using High Angular Resolution Diffusion Imaging (HARDI; 61 directions, *b*-value 1500 mm^3^). Following constrained spherical deconvolution whole brain tractography, two independent raters isolated the SM. Each output was checked, examined and cleaned for extraneous streamlines inconsistent with known anatomy of the tract by the rater and a neuroanatomist. A second neuroanatomist assessed tracts for face validity. The SM was reconstructed with excellent inter-rater reliability for dimensions and diffusion metrics. Gender had no effect on the dimensions or diffusion metrics, however radial diffusivity (RD) showed a positive correlation with age. Reliable identification and quantification of diffusion metrics of the SM invites further exploration of this key habenula linked structure in neuropsychiatric disorders such as depression, anxiety, chronic pain and addiction. The accurate anatomical localization of the SM may also aid preoperative stereotactic localization of the tract for deep brain stimulation (DBS) treatment.

## Introduction

The dorsal diencephalic conduction system is a pathway that transmits information from the cognitive-emotional forebrain to the regulatory midbrain areas (Sutherland, 1982). This highly conserved system (Beretta et al., 2012) gathers fibers from diverse frontal areas including the septal nuclei (pleasure and motivation), dorsal anterior cingulate (reward-based decision making), lateral hypothalamus (arousal and pain) and basal ganglia (motor and behavioral control). After forming a single unidirectional tract, these fibers project to the habenulae, a set of relay nuclei at the dorso-caudal end of the thalamus (Parent et al., 1981; Geisler and Trimble, 2008). The habenula in turn projects down through the fasciculus retroflexus influencing midbrain monoamines of the raphe nuclei and ventral tegmental area. Through this system the frontolimbic areas can influence midbrain and whole brain monoaminergic tone. The single unidirectional tract connecting the fronto-basal areas to the habenula is called the stria medullaris (SM).

**Figure 1:**
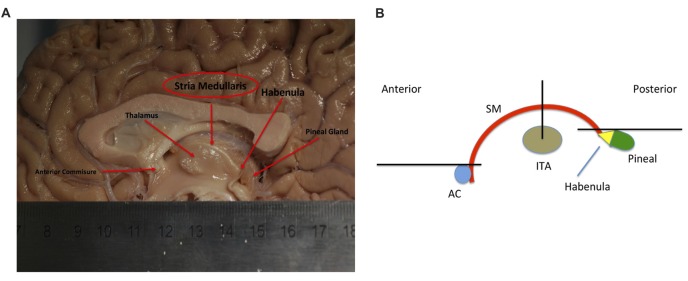
**(A)** Showing a mid-sagittal slice of a donor cadaver brain. The SM and its boundaries are shown. Note particularly how the SM curves over the inter-thalamic adhesion. Also note, the length of the arch is approximately 30 mm. Image courtesy of the Department of Anatomy, Trinity College Dublin. **(B)** Schematic diagram showing the SM (in red) and landmarks for gate placement. Three gates were placed using the anterior commissure, pineal gland and thalamus as landmarks. Note particularly the line drawn horizontally from the uppermost point of the pineal. Where this intersects the SM represents the most caudal aspect of our reconstructed tract. This point was chosen for reasons of consistency. The habenula was not always reliably seen on MRI and the pineal was chosen as the closest anatomical landmark. As such the protocol routinely cuts the SM slightly short, stopping just before its insertion into the habenula. AC, Anterior Commissure; ITA, Inter-thalamic adhesion, SM, Stria Medullaris.

The SM (or SM thalami) is a white matter epithalamic tract (Figure 1). It emerges just caudal to the anterior commissure as a solitary tract that arches both dorsally and caudally over the inter-thalamic adhesion. It terminates at both lateral and medial habenular nuclei (Parent et al., 1981; Geisler and Trimble, 2008; Ahumada-Galleguillos et al., 2017). The habenular nuclei receive input chiefly through SM (Herkenham and Nauta, 1977). The area of the habenula occupied by fibers that enter the habenula through the SM is considerably larger in humans compared to other species. Post mortem studies suggest that the human SM covers about 30% of the entire cross-sectional area of the habenula, compared to only 12% in rodents (Diaz et al., 2011; Ahumada-Galleguillos et al., 2017). This most likely reflects the increased connectivity of the enlarged human forebrain to the habenula, consistent with its function in relaying information from frontal to midbrain regions.

The habenula outputs either directly or indirectly to midbrain monoaminergic nuclei to affect the release of serotonin (Kalén et al., 1985), dopamine (Gruber et al., 2007) and norepinephrine (Quina et al., 2015). In general, habenula activation reduces monoaminergic output (Boulos et al., 2017). Using human neuroimaging, the habenula has been implicated in aversive decision making (Lawson et al., 2014; Hennigan et al., 2015), reward pathways (Salas et al., 2010), mood (Morris et al., 1999; Ely et al., 2016) and pain (Shelton et al., 2012). Habenula changes have also been found in disorders such as depression (Lawson et al., 2017; Liu et al., 2017) and chronic pain (Erpelding et al., 2014). The unidirectional flow of information from the limbic frontal regions through the SM to the habenula and the apparent direct relationship of habenula activity to neurotransmitter release invite study of the SM in neuropsychiatric disorders such as depression, bipolar disorder, schizophrenia, chronic pain and addiction our (Fakhoury, 2017). Due to its strategic location, the SM may provide information on potential problems upstream to the habenula. However, no study has investigated the role of this key structure in disease. This is due to a lack of techniques to assess this white matter tract *in vivo* in humans.

Recent advances in diffusion-weighted magnetic resonance imaging (DWI) tractography have allowed identification of white matter tracts with ever-greater precision (Johansen-Berg and Rushworth, 2009; Farquharson et al., 2013). This technique has been particularly useful for identifying large white matter tracts such as the superior longitudinal fasciculus, inferior longitudinal fasciculus and corpus callosum (Mangin et al., 2013). However, parsing out the smaller and more tortuous tracts of the limbic system using tractography can be complicated by methodological difficulties such as resolution issues and crossing fibers (Mori and Aggarwal, 2014). Newer, more advanced technology is aiding with these endeavors. Increasing MR field strengths and the use of faster acquisition sequences can reduce scan times while increasing the signal to noise ratio (Soares et al., 2013). Preprocessing developments such as head motion, distortion and free water correction techniques can also increase the signal to noise ratio (Tournier et al., 2011). Diffusion models such as constrained spherical deconvolution (CSD; Jeurissen et al., 2011), diffusion spectrum imaging (Wedeen et al., 2008) and Q-Ball imaging (Tuch, 2004) are allowing previously hidden crossing, diverging and kissing fibers to be explored. Such developments are facilitating accurate reconstruction of limbic tracts such as the fornix (Christiansen et al., 2017), ventral amygdalofugal pathway (Kamali et al., 2016), stria terminalis (Kamali et al., 2015) and medial forebrain bundle (Hana et al., 2015). Tract diffusion metrics such as fractional anisotropy (FA) and radial diffusivity (RD) may provide information on the condition of limbic white matter tracts in disease (Metzler-Baddeley et al., 2012; Oishi et al., 2012).

The SM as a discrete white matter tract originates just behind the anterior commissure and terminates at the habenula. It does not deviate or branch between these two fixed points. This characteristic makes it a good potential candidate for tractography. However, the SM is particularly challenging to accurately render with DWI tractography. It is short, thin and highly curved (180 degree turn along its course) making it methodologically tricky for DWI tractography to process. It lies deep within the brain making it difficult to isolate from the multiple surrounding white matter structures. Finally, the SM and its termination, the habenula, are inconsistently seen on standard T1/T2 MRI due to being “missed” between slices (Strotmann et al., 2014; Kochanski et al., 2016).

Accurately imaging the SM using tractography could facilitate research into the neuropathology of psychiatric, addiction and pain disorders through the analysis of tract diffusion metrics. Furthermore, its pivotal role in integrating diverse basal forebrain limbic regions into a single tract and relaying directly with the habenula makes the SM a target for deep brain stimulation (DBS) in the treatment of severe depression (Sartorius et al., 2010; Kiening and Sartorius, 2013). Accurately mapping the trajectory may aid the perioperative localization of the tract in stereotactic surgery. This study aimed to reconstruct the SM trajectory in 50 healthy individuals with the limitations of DWI tractography of this region in mind. The signal to noise ratio was maximized by careful preprocessing of the data including signal drift, head motion, eddy current and EPI distortion correction. High Angular Resolution Diffusion Imaging (HARDI) data was acquired and a CSD model was used to create tracts of the entire brain. This algorithm has been shown to overcome some of the known difficulties and shortcomings of the diffusion tensor model and has the ability to model regions with complex fiber orientations such as multiple or crossing fibers within a voxel, allowing more convoluted tracts to be accurately explored. Due to the SM’s size and highly curved shape, a high angle threshold and small step size was used. Neuroanatomical expertise was also involved at all stages of protocol design. Reliability between raters was measured to evaluate the consistency of the protocol and age and sex differences of the SM were assessed using diffusion metrics. As such we present here a novel and reliable method for reconstructing the SM *in vivo* using constrained spherical deconvolution based deterministic tractography.

## Materials and Methods

### Participants

Fifty participants (25 female) were enrolled through an active database of willing participants as part of the REDEEM (Research in Depression: Endocrinology, Epigenetics and neuroiMaging) study at Trinity College Dublin. The mean age and standard deviation (SD) was 29.58 (SD 13.03) with ages of females 29.85 (SD 11.93) and males 29.31 (SD 14.28). Exclusion criteria for study entry included contraindications to MRI, history of illicit substance abuse, head trauma, and any significant medical or psychiatric illness. All participants underwent a Structured Clinical Interview (SCID) for DSM IV, Hamilton Depression Rating Scale (HAM-D; Hamilton, 1960) and Hamilton Anxiety Rating Scale (HAM-A; Hamilton, 1959) administered by a psychiatrist at TCIN to further exclude psychiatric pathology. Subjects were required to have no active or previous SCID diagnosis, a HAM-D score of 8 and below and a HAM-A score of 13 and below.

This study was carried out in accordance with the recommendations of the Tallaght Hospital/St. James Hospital Joint Research Ethics Committee with written informed consent from all subjects. All subjects gave written informed consent in accordance with the Declaration of Helsinki. The protocol was approved by the Tallaght Hospital/St. James Hospital Joint Research Ethics Committee.

### MRI Acquisition

All data was acquired on a Philips (Best, Netherlands) Intera Achieva 3.0 Tesla MR system (32-channel head coil) at Trinity College Institute of Neuroscience, Dublin. One-hundred and eighty axial high-resolution T1-weighted anatomical images (T1W-IR1150 sequence, TE = 3.8 ms, TR = 8.4 ms, FOV 230 mm, 0.898 × 0.898 mm^2^, in-plane resolution, slice thickness 0.9 mm, flip angle alpha = 8°). Whole brain, HARDI (Jones and Leemans, 2011; Jones et al., 2013) was acquired using a spin-echo echo-planar imaging (SE EPI) pulse sequence (TE = 52 ms, TR = 11,260 ms, flip angle alpha = 90°), FOV 224 mm, 60 axial slices, 2 mm^3^ isotropic voxels, *b*-value = 1500 s mm^−2^ in 61 non-collinear gradient directions. A single non-diffusion-weighted b0 image was also obtained.

### DWI Preprocessing and Whole Brain Tractography

DWI preprocessing involved the following steps in *ExploreDTI*^1^ (Leemans et al., 2009), a free MATLAB^2^ based graphical toolbox for diffusion MRI preprocessing and tractography. Diffusion preprocessing involved the following steps: (a) exporting and standardizing diffusion output files; (b) signal drift correction via linear and quadratic correction (Vos et al., 2017); (c) Gibbs Ringing artifact correction (Perrone et al., 2015); (d) orientation/directionality checks via manual glyph inspection; (e) head motion and eddy current artifact correction using rigid body and affine registration respectively to the non diffusion weighted b0 image (Soares et al., 2013); and (f) EPI deformation correction via affine registration to the T1 image (Irfanoglu et al., 2012). These operations were performed using the latest version toolbox plugins directly within *ExploreDTI*.

**Figure 2:**
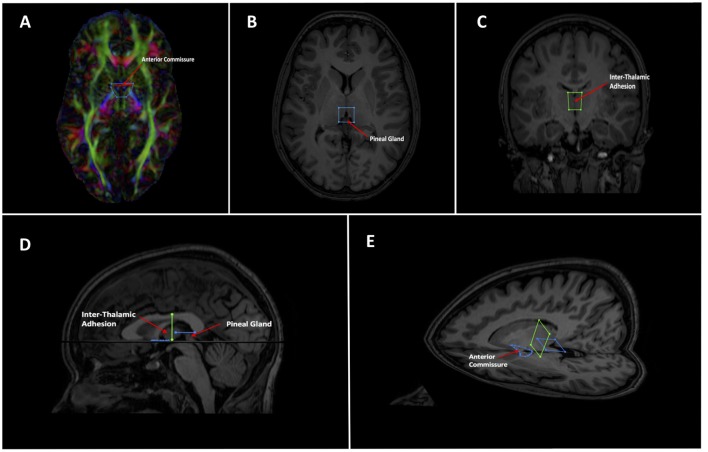
Showing initial gate placement. **(A)** Anterior SEED gate placement: the most superior point of the anterior commissure is found on a First eigenvector fractional anisotropy (FEFA) axial DWI slice. The anterior boundary of the SEED gate is placed just anterior to the commissure and the gate is extended broadly posteriorly past the columns of the fornix. The anterior commissure is labeled. **(B)** Posterior SEED gate placement: In an axial T1 image, the slice *above* the most superior visible part of the pineal gland is chosen. The anterior boundary of the SEED gate is placed approximately at the mid thalamus and extended to where the mid pineal is found on the slice below. The gate should extend laterally to cover the approximate stalks of the pineal gland. Note that the pineal *is visible* in this image, as this is taken to show the pineal for localization purposes. The pineal is labeled. **(C)** Mid AND gate placement: the mid-point of the thalamus (using the inter-thalamic adhesion if present) is found on a coronal T1 image slice. The superior boundary of the AND gate is placed above the thalamus at the lower level of the fornix and extended to the lower thalamus, making sure to encompass the inter-thalamic adhesion if present. The gate should be wide enough to cover most of the “curve” of the thalamus. The inter-thalamic adhesion is labeled. **(D)** Sagittal and **(E)** axial-sagittal views of the initial SEED and AND gates. The anatomical fixed points for gate placement: anterior commissure, inter-thalamic adhesion and pineal gland are labeled.

Tractography of the whole brain was generated in *ExploreDTI* using constrained spherical deconvolution (as a recursive calibration of the response function; Jeurissen et al., 2011). Whole brain seeding was used with seed voxel sizes of 2 mm^3^ and a fiber orientation distribution threshold of 0.1. Whole brain tractography was achieved through multiple random seed placements of one seed per voxel (Jones, 2010; Jones and Leemans, 2011). Two separate whole brain tractography files were generated on each subject. First, a sparser one with fewer and less dense streamlines was created to easily scout out the general region of the SM—low fidelity tracts. This was only used for initial gate placement (Figure 2) and to identify larger structures that needed to be excluded e.g., the fornix. Subsequently, a more complex tract with a higher density of streamlines was created to reconstruct the tract at the latter stages—high fidelity tracts. The step size was set at both 1 mm and 0.5 mm and the maximum angle threshold was set at 45° and 89°, respectively for low and high fidelity whole brain tracts. All whole brain tract lengths were set between 10–500 mm. The lower threshold was chosen as we assumed the SM would be likely greater than 10 mm (around 30 mm from our own anatomical measurements). The higher threshold was chosen to faithfully reproduce larger known tracts that surround the SM region, such as the fornix (Metzler-Baddeley et al., 2013), stria terminalis (Kamali et al., 2015), thalamocortical radiations (Kubota et al., 2013) and superior thalamic peduncle (Meola et al., 2015). These tracts were subsequently removed with NOT gates. No other tractography stopping criteria apart from angle threshold, FOD threshold and tract length were used.

**Figure 3:**
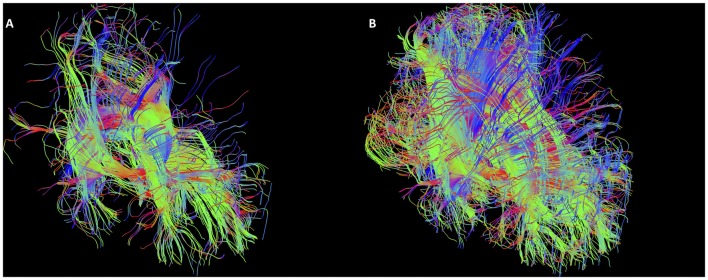
Initial low fidelity **(A)** and high fidelity **(B)** tracts on the same subject. As seen here, the tracts generated from the initial three gates using the high fidelity tracts are too complex to easily discriminate known white matter bundles. For example, the crus of the fornix can be easily seen in **(A)**, but not in **(B)**. Low fidelity tracts allowed us to place NOT gates appropriately to remove known white matter tracts (see Figure 4). Following removal of these extraneous fibers, the low fidelity tracts were discarded. The NOT gates were then used with the high fidelity tracts to generate the SM.

As mentioned, the low fidelity tracts were used as scout tracts to aid initial gate placement and early cleanup. During our initial investigations into tractography of the SM, we found that having whole brain tracts with a small step size and high angle created overly complex images due to a profuseness of streamlines and multiple interweaving tracts (see Figure 3 for examples of low and high fidelity tracts prior to cleanup). This created problems when trying to exclude the larger surrounding tracts mentioned above. As a result, we decided that using less complex whole brain tracts with a larger step size and lower angle greatly aided our initial virtual “dissection” process. Once the larger known tracts were excluded and we visualized the region where we suspected the SM to be present, we saved the cleanup gates and applied them to the high fidelity tracts. High fidelity gates with small step size and a high angle were necessary to accurately capture the highly curved anatomy of the SM.

### Gate Placement

Following consultation with the Department of Anatomy at Trinity College Dublin, a Boolean logic protocol was designed to isolate the SM using AND and OR gates within *ExploreDTI*. Emphasis was given towards gates that: (a) included the maximum possible number of SM streamlines; (b) were positioned around landmarks clearly identifiable on DW or T1 images; and (c) that would consistently capture at least some section of the SM. Initial gate consensus consisted of two OR gates in the axial plane at the agreed anticipated anterior and posterior margins of the SM and an AND gate in the coronal plane as the SM arcs over the inter-thalamic adhesion. Three AND gates would generate the same final result, however in our exploratory investigations and rater training session, two OR gates were selected at the anterior and posterior margins, so our protocol continued to use this for consistency.

As the SM neurons merge into a solitary tract just behind the anterior commissure (Figure 1), the uppermost section of the commissure as seen on DWI was used as the primary landmark for placement of the anterior axial OR gate (Figure 2A). The SM terminates posteriorly in both habenulae (Figure 1). This tiny structure is not seen consistently in either DW or T1 images (Lawson et al., 2013). The habenulae bilaterally are within the stalks of the pineal gland, a structure consistently seen on T1 MRI. However, the position of the pineal relative to its stalk as seen on MRI depends on the size of the gland and on how the pineal “hangs” within the quadrigeminal cistern. To reliably include part of the SM tract, the superior most point of the pineal on T1 imaging was established as the only consistent primary landmark for placement of the posterior OR gate (Figures 1, 2B). Occasionally this resulted in a shortened SM that omitted the posterior end. Finally, the SM always arcs between both thalami and above the inter-thalamic adhesion if present (Figure 1). The inter-thalamic adhesion is not present in 20%–30% of individuals (Allen and Gorski, 1991); as such the inner half of both thalami on coronal T1 imaging was used as the primary landmark for the final AND gate (Figure 2C). Gate placement is described in detail in Figure 2.

**Figure 4:**
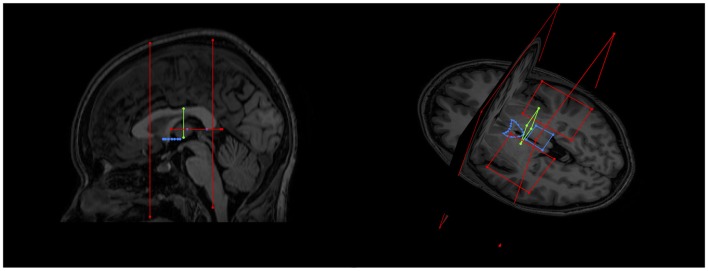
Examples of initial NOT gates on sagittal and axial-coronal views. Two coronal whole brain NOT gates were placed with generous margins anterior and posterior to the area where the SM was thought to be. Two axial NOT gates removed the majority of fornix fibers by isolating the crus of the fornix bilaterally.

**Figure 5:**
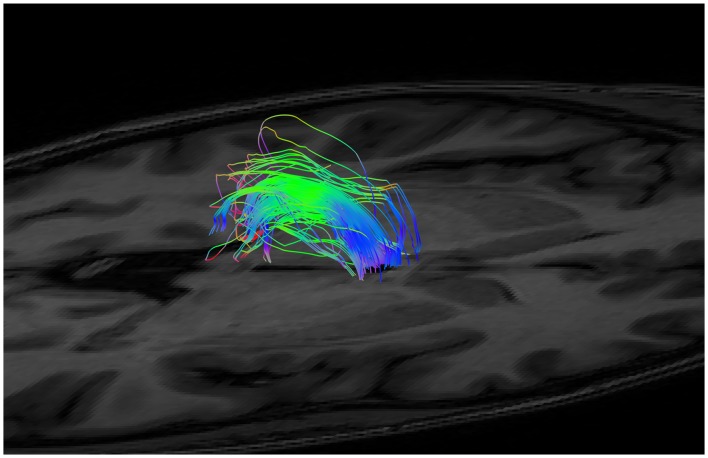
An example of generated tracts post segmentation and splitting (i.e., isolation of the fibers between the anterior and posterior margins only), but prior to neuroanatomical cleanup.

Following an initial training period on a different dataset of 10 subjects, two raters placed the gates on DW and T1 MR images of 50 subjects. Raters were blind to age and sex of the subjects. The gates were applied to the low fidelity scout whole brain tracts to generate streamlines of interest encompassing the SM. Larger obviously non-SM tracts (most notably the fornix) were excluded using NOT gates; see Figure 4 for details of typical NOT gates. The remaining streamlines were split and segmented (using the Splitter and Segmentation tools in *ExploreDTI*) to include all fibers that only travel between the three gates yet eliminate those sections of the streamlines peripheral to the anterior and posterior gates (see Figure 5). Once satisfied that the SM could be visualized within the section, the high fidelity whole brain tracts were reloaded with the gates in place. The low fidelity scout tracts were then discarded. With the gates now in place, the high fidelity whole brain tracts could be used to “dissect” out the region around the SM.

### Cleanup

The next step required that streamlines not associated with the SM be removed. Cleanup was achieved with each rater and a neuroanatomist examining every subject together, collectively excluding streamlines not considered SM fibers using NOT gates. Criteria for exclusion included streamlines deviating from the known arch shape of the SM and streamlines consistent with known adjacent tracts. To address potential bias in the same subject between raters, the neuroanatomist was blind to the identity of each subject. These NOT gates were variable according to each subject and depended on the individual neuroanatomy of the region. However, commonly excluded streamlines included fibers from the body of the fornix, the stria terminalis, the median forebrain bundle, superior thalamic peduncle, and various inter-thalamic connections. A second neuroanatomist reviewed a random subsample of 25 tracts from both raters to check face validity of the SM (looking at start and end points, the arch shape, the tract’s relationship to other structures on MRI images (thalamus, fornix, anterior/posterior commissures and third ventricle) as well as the overall general impression of the tract. The second neuroanatomist also checked both raters’ tracts simultaneously to investigate if they were occupying approximately the same space. This was considered a sufficient subsample for quality control.

Tract statistics including dimensions (length and volume), standard diffusion measures such as FA, mean diffusivity (MD), axial diffusivity (AD) and RD and Westin diffusion measures [Spherical Diffusion (C_S_), Linear Diffusion (C_L_) and Planar Diffusion (C_P_)] were calculated on each tract using *ExploreDTI’s* built in tract statistics plugin.

### Estimated Total Intracranial Volume and Statistical Analysis

Estimated Total Intracranial Volume (eTIV) was calculated from the T1 images using the *Freesurfer 6.0* image analysis suite^3^ (Fischl, 2012). The technical details of this procedure are described elsewhere (Buckner et al., 2004; Voevodskaya et al., 2014; Sargolzaei et al., 2015).

### Statistics

A comparison of reliability between each independent rater was performed using interclass correlation coefficient (ICC) analysis using SPSS24^4^ for the following measurements: dimensional measures, SM length and tract volume, standard diffusion metrics, FA, MD, AD and RD and Westin diffusion measures, C_S,_ C_L_ and C_P_. Rater measures were averaged to represent a mean DWI measure for each metric and the effect of gender on each diffusion metric was determined using independent Analysis of Covariance (ANCOVA) in SPSS correcting for age and eTIV. Finally, partial correlation analyses were performed to examine the effect of age on each diffusion metric when controlling for gender and eTIV.

## Results

**Figure 6:**
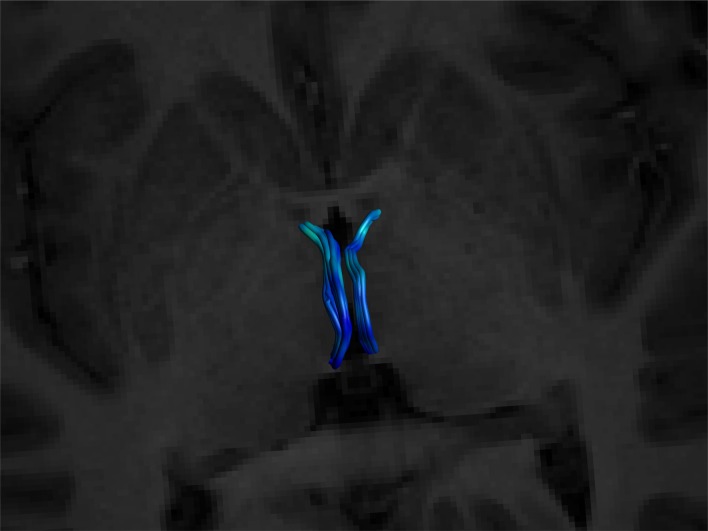
Superior view of a bilateral SM on an axial T1 plane. Note its posterior insertion into the high signal intensity regions at the posterior thalamus, consistent with white matter. Male, aged 27.

**Figure 7:**
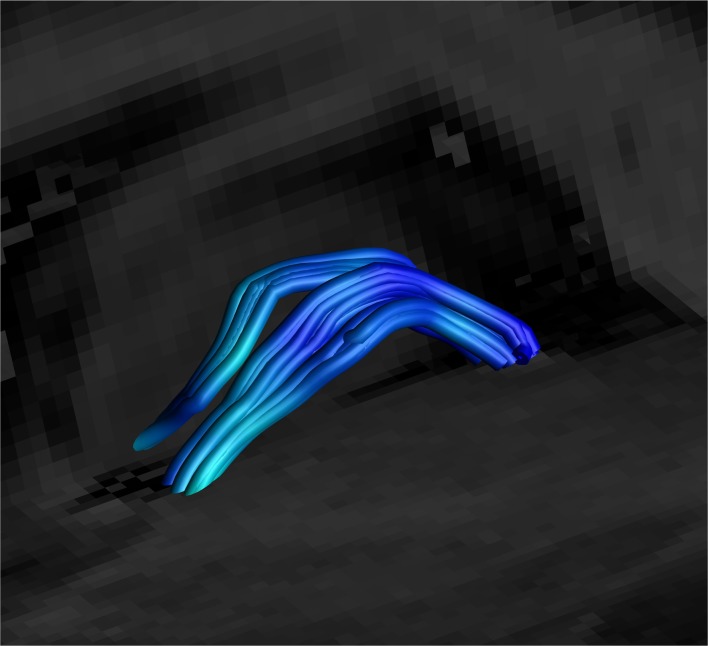
Close up side view of a SM on sagittal and coronal T1 planes, clearly showing the arc of the tract. Female, aged 31.

**Figure 8:**
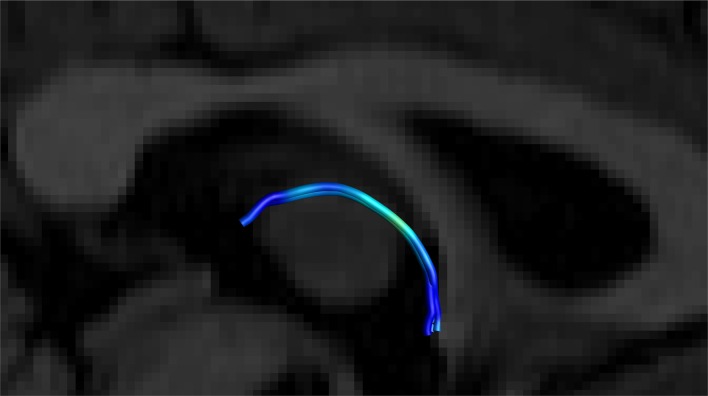
Close up side view of a single right-sided SM on a T1 sagittal plane (the left side is hidden behind the T1 sagittal plane) showing the stria arching over the inter-thalamic adhesion. Male, aged 40.

**Figure 9:**
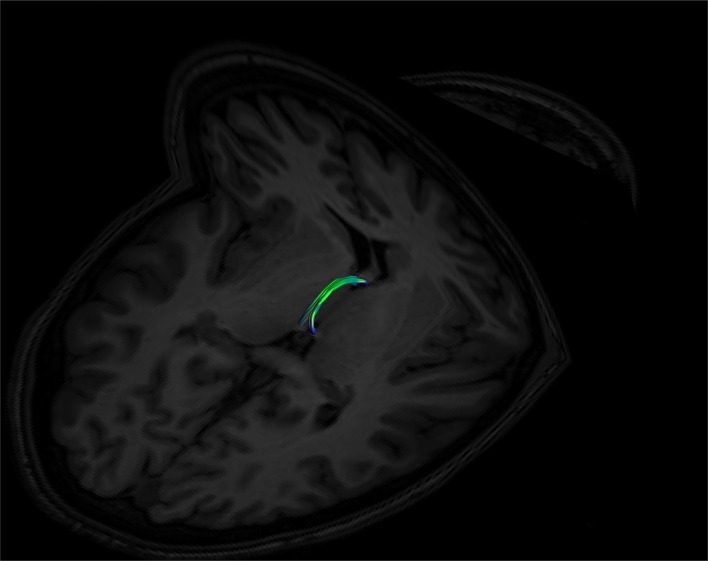
An example of a left and right fused SM on axial and coronal T1 image planes. The left and right sides are fused in the middle of the tract. Note its posterior insertion into the high signal intensity regions at the posterior thalamus, consistent with white matter. Female, aged 22.

**Figure 10:**
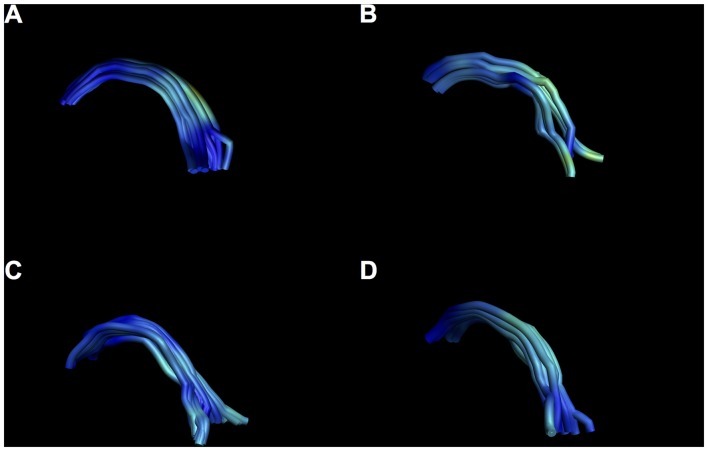
Examples of intersubject variability of SM reconstruction. Note that all tracts have the characteristic arch shape, but some show a more bilateral divide than others. **(A)** Male, aged 35; **(B)** female, aged 25; **(C)** male aged 40; **(D)** female, aged 37.

The SM was generated in 50 subjects by two raters and reconstructed consistently in every subject (Table 1). Even though the SM can be considered a bilateral structure, both halves unite into a single tract as it makes its way from front to back. As such it was not possible to reliably separate out the left from right SM using tractography. These results are presented as a single *total bilateral* (i.e., left and right SM combined) tract. Examples of generated SM are found in Figures 6–10. Figure 10 demonstrates some of the inter-subject variability seen in the tract. A video showing a rotating SM to demonstrate the structure in 3D is included in the supplementary section. The second neuroanatomist had concerns with one generated SM (the arch appeared flattened on inspection), which was redone afresh and cleaned as per protocol. Both neuroanatomists and rater agreed with the changes.

**Table 1:** Interclass correlation coefficient between each rater for dimension and diffusion measurements of the stria medullaris (SM).

	Rater 1 mean	Rater 2 mean	ICC	95% CI	Gender effect (*p*-value)	Age correlation (*p*, *r*-value)
**Dimensions**						
Length (mm)	29.8	30.3	0.86	0.77–0.92	0.13	0.9, −0.02
Volume (mm^3^)	129	131	0.89	0.82–0.94	0.63	0.9, +0.02
**Diffusion metrics**						
FA	0.253	0.251	0.92	0.86–0.95	0.67	0.08, −0.25
MD	0.00112	0.001113	0.96	0.92–0.94	0.54	0.05, +0.28
AD	0.00141	0.00141	0.96	0.93–0.98	0.58	0.1, +0.25
RD	0.00098	0.00099	0.95	0.92–0.97	0.52	0.04, +0.30
C_L_	0.243	0.241	0.93	0.87–0.96	0.93	0.37, −0.13
C_P_	0.145	0.145	0.97	0.95–0.98	0.47	0.19, −0.19
C_S_	0.612	0.615	0.93	0.88–0.96	0.59	0.06, +0.28

*The effect of gender on each measure following ANCOVA, correcting for age and eTIV is shown. The effect of age on each measure following partial correlation analysis correcting for sex and eTIV is also shown. AD, Axial Diffusivity; ANCOVA, Analysis of Covariance; CL, Linear Diffusion; CI, Confident Interval; CP, Planar Diffusion; CS, Spherical Diffusion; FA, Fractional Anisotropy; ICC, Inter-rater correlation coefficient; MD, Mean Diffusivity; RD, Radial Diffusivity*.

**Figure 11:**
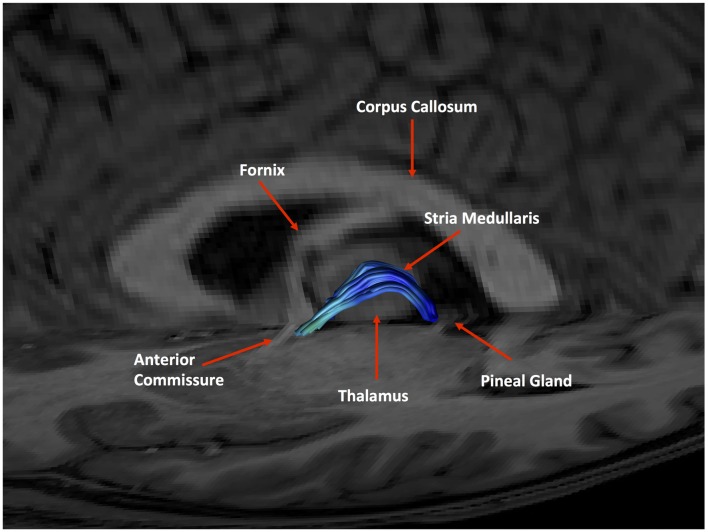
An example of a generated tract in relation to anatomical landmarks. The anterior commissure, thalamus and pineal gland are labeled.

As expected, the structure arched dorso-caudally until about mid-thalamus and then continued ventro-caudally as it neared the habenula. Where there was an inter-thalamic adhesion, the SM arched dorsal to this. A generated tract in relation to anatomical landmarks is shown in Figure 11. Our current protocol captures the SM only from the anterior commissure rostrally to where the arch reaches the level of the uppermost point of the pineal gland as it continues to arch ventrally (see Figure 1, and for more details see “Materials and Methods” section). This landmark was chosen for anatomical and imaging consistency, even though it routinely resulted in a slightly shorted SM, omitting the caudal most aspect of the tract where it meets the habenula. The mean lengths of the tract were 29.8 mm and 30.3 mm for Rater 1 and 2, respectively. The mean volumes of the SM were 129 mm^3^ and 131 mm^3^ for Rater 1 and 2. Inter-subject variability is shown in Table 2. There is no reported SM length in the literature to our knowledge. However, these lengths correspond with measured SM lengths in eight cadaveric brains by the Anatomy Department at Trinity College Dublin of between 28 mm and 35 mm.

**Table 2:** Inter-subject SM dimension variability.

			95% Confidence interval	
	Mean volume (mm^3^)	Standard error	Lower bound	Upper bound
Female (*N* = 25)	125.097	12.442	100.053	150.142
Male (*N* = 25)	134.844	12.442	109.801	159.889
Total	129.971	7.506	114.862	145.079
	**Mean length (mm)**	**Standard error**	**Lower bound**	**Upper bound**
Female (*N* = 25)	29.124	0.733	27.647	30.623
Male (*N* = 25)	30.935	0.733	29.459	32.412
Total	30.031	0.442	29.129	30.92

* Volumes, lengths and standard errors are shown. The upper and lower limits of data within the 95% confidence interval are also shown*.

Good inter-rater reliability was demonstrated for each measurement (mean ICC 0.91 over all measures). There were no significant differences between males and females for any DWI metric examined. Partial correlation analyses between diffusion metrics and age identified a positive correlation for RD only (*p* = 0.038, *r* = 0.3) when correcting for gender and eTIV. No other metric showed a significant correlation with age (*p* < 0.05); refer to Tables 1, 2 for results.

## Discussion

This study describes a reliable technique for reconstructing the functionally important SM using diffusion-weighted tractography. The technique demonstrates excellent dimensional inter-rater reliability between two independent raters and face validity from a second independent neuroanatomist. Tract metrics also showed excellent inter-rater reliability with high ICCs for dimensional measures (length and volume) and diffusion metrics. The mean tract length were 29.8 mm (Rater 1) and 30.3 mm (Rater 2) and correspond to observed tract lengths of between 28 mm and 35 mm in cadaveric brains. Of note, there was no difference in SM dimensions or diffusion metrics between males and females.

Only one SM diffusion metric changed with age. RD, a measure of diffusivity perpendicular to the fiber tract orientation increased with age. AD, a measure of diffusivity parallel to the tract, showed no change with age. FA showed no change with age. This is a measure of the relative diffusivity along the bundle. Often in aging white matter tracts, FA decreases as RD increases (Bennett et al., 2010). This is due to the relatively greater change of diffusivity along the tract compared to across it. This is not the case in our SM reconstructions, where only RD increases. The increase in RD with age is consistent with other studies reporting an increase across multiple white matter brain regions (Hsu et al., 2008) and particularly in limbic areas (Kumar et al., 2013). This RD change may reflect deteriorations in myelin with age (Song et al., 2002; Wu et al., 2011), and myelin loss is considered a normal part of healthy aging (Peters, 2002). Dorsal diencephalic conduction system input changes through an aging SM may manifest as overall changes in mood, hedonic states and motivation that occur with age (Prince et al., 1999; Harada et al., 2013; Sutin et al., 2013).

Only one previous study has investigated the SM using diffusion-weighted imaging (Kochanski et al., 2016). This study used a probabilistic local approach in five pre-surgical patients defining the estimated site of the habenula as a sole seed point. Using a single probabilistic seed point with a local approach may have limitations in capturing the known gross anatomy of the SM. Indeed the tracts in this study do not arch over the inter-thalamic adhesion, unlike our SM tracts. A local seed based approach is susceptible to errors as the tract propagates (Reisert et al., 2011; Anastasopoulos et al., 2014). We used three anatomically guided gates to accurately identify the known arch-like shape, followed by careful cleaning according to known boundary structures. This anatomically guided approach on global (whole brain) tracts allowed us to predefine our tract start (anterior) and end (posterior) points for consistency. Having tracts with defined boundaries will allow easy comparison between individuals for future studies. Using a third gate across the thalamus and above the inter-thalamic adhesion allowed us to focus solely on streamlines that corresponded to the known anatomical arch of the SM and reduced the likelihood of generating deviant tracts. We believe our approach more accurately describes the trajectory of the SM and could be useful in conjunction with standard structural imaging in perioperative stereotactic localization of the SM for DBS. Furthermore, the habenula is a small structure of approximately 30–36 mm^3^ (Savitz et al., 2011; Lawson et al., 2013) and may not be accurately identified on standard MRI especially at 3T (Strotmann et al., 2014). We felt it more appropriate to use an imaging fixed point at the pineal to optimize consistency and reliability. Our present study has some other advantages including a higher number of subjects (50), healthy controls instead of patients, prior neuroanatomical planning and face validity checks from a second neuroanatomist.

### Strengths and Limitations

Deterministic tractography is an experimental method that is subject to some technical challenges (Jones, 2008). There are known issues involving curving and kissing tracts. The SM is highly curved and turns 180° in a relatively tight space. This was addressed by reconstructing the SM using high angle (89°), small step-size (0.5 mm) whole brain tracts. The SM lies deep within the center of the brain, surrounded by and touching other white matter bundles; the fornix, stria terminalis and various thalamic connections. CSD was chosen as the most appropriate tractography technique due to its superior ability at detecting complex crossing and kissing fibers in a recent analysis of fiber estimation approaches (Wilkins et al., 2015). As with all diffusion-weighted reconstruction, the generated SM only describes the diffusivity in the region of interest and it is assumed that this corresponds to an actual tract. The length of our generated SM (30 mm) approximates to the known length of the SM. Also, a subsample of images (50%) was presented to a second neuroanatomist to check face validity of the results. To address this further we plan to systematically measure the length and curvature of a number of cadaveric SMs to determine baseline dimensions of this tract for comparison.

An experienced neuroanatomist with expertise of the region aided the rater during cleanup. We felt this represented the most valid approach for removing extraneous streamlines. As the protocol was designed to encompass as much of the SM as possible in a consistent manner, the initial gates were overly broad. This resulted in a large amount of cleanup. The average time of cleanup (both rater and neuroanatomist working together) was estimated at about 20 min per subject. It is appreciated that this approach may not be available or practical for some centers. However, with adequate neuroanatomical training, this limitation may be overcome and a researcher/technician experienced with *ExploreDTI* and the complex regional anatomy could single-handedly undertake the entire process.

One limitation is that the posterior limit of the tract was occasionally clipped due to the to position of the gate at the pineal gland. The terminus of the SM anatomically is the habenula, which lies within the stalks of the pineal. The habenulae/pineal stalks are inconsistently seen on standard T1 images (Lawson et al., 2013), and as such the pineal was chosen as a marker. However, we found during our initial exploration of potential gates that only a gate placed just above the level of the most superior point of the pineal guaranteed inclusion of the SM. Our reconstructed tracts are therefore slightly shorter than anatomical ones. Higher resolution T1 imaging showing the pineal stalk may address this problem for future studies.

The mean FA (0.251) calculated for our tract was low compared to other limbic tracts e.g., stria terminalis (FA = 0.37; Kwon et al., 2011), mammillothalamic tract (FA = 0.38; Kwon et al., 2010), pre-commissural (FA = 0.341) and post commissural fornix (FA = 0.359; Yeo et al., 2013). This could be explained by the fact the SM exists as a small solitary tract within the third ventricle surrounded by cerebrospinal fluid (CSF) on three sides for most of its course. The diameter of the SM as measured through our initial cadaveric measurements was between 1.5 mm and 2.5 mm. As our voxel size was 2 mm^3^, it is probable that partial volume effects where both CSF and SM mixed within voxels were present in this region. Partial voluming from inclusion of CSF has been shown to collapse FA (Alexander et al., 2001). FA values of below 0.25 in otherwise healthy adults with normal appearing white matter may reflect this effect rather than changes in white matter coherence (Pfefferbaum and Sullivan, 2003). We plan to address this in future research by decreasing our voxel size to 1 mm^3^ and also through using free water correction methods (Pasternak et al., 2009).

### Implications

As the first component of the dorsal diencephalic conduction system, the SM is fundamental in the flow of information from frontal-limbic regions to key midbrain monoaminergic nuclei (Sutherland, 1982). The habenula has been shown to be involved in aversive conditioning, reward pathways (Boulos et al., 2017) and in the pathophysiology of several psychiatric conditions (Fakhoury, 2017). The DCCS seems to be particularly relevant in negative reward prediction errors, or “disappointment”, where failure to receive an expected reward integrates cognitive and emotional inputs through the habenula to affect a depressive behavioral response (Kaye and Ross, 2017).

In particular, changes in structure (Savitz et al., 2011; Schmidt et al., 2017) and function (Furman and Gotlib, 2016; Lawson et al., 2017) of the habenula have been shown in depression. This makes sense considering the DDCS’s pivotal role in mediating serotonergic tone. Depressive symptoms, in particular anhedonia and motivation, have been associated with disrupted habenula function (Lawson et al., 2017; Liu et al., 2017). This reflects its role in connecting the basal septal pleasure areas and midbrain monoaminergic response. However, the habenula’s main afferent input from these frontal regions, the SM, has not been a focus of clinical study, mainly due to the difficulties in assessing the tract *in vivo*. Using our approach, it may be possible to use diffusion metrics to investigate the SM in a wide range of neuropsychiatric conditions such as depression and anxiety (serotonin), schizophrenia and addictions (dopamine) and pain (noradrenaline). Changes in afferent habenula inputs may be reflected in diffusion metric changes, focusing attention on regions upstream to the DCCS such as the septal pleasure areas. Future research using higher resolution imaging may also reveal the lateral and medial habenular components (Strotmann et al., 2014) and their discrete afferents via the SM using diffusion connectomics, furthering our understanding of the complex and distinct connections between the forebrain and midbrain.

The SM has long been suggested as a potential DBS target in treatment-resistant depression (Blander and Wise, 1989; Sartorius and Henn, 2007; Sartorius et al., 2010; Kiening and Sartorius, 2013; Kochanski et al., 2016). This is due its key role in integrating diverse basal forebrain into a solitary white matter bundle prior to processing in the habenula. It has been attempted in one patient who showed remarkable improvement from intractable depression following high frequency stimulation of the SM (Sartorius et al., 2010). Our tractography approach may aid future 3D stereotactic localization of the SM prior to electrode placement in such a patient, as the habenulae and SM are inconsistently seen on standard structural MRI (Lawson et al., 2013; Kochanski et al., 2016). As well as providing another layer of anatomical certainty in combination with standard imaging during electrode insertion, SM diffusion metrics may also offer insights into the microstructural integrity of the tract pre and post treatment.

This study establishes a novel and reliable method for reconstructing the SM using deterministic diffusion-weighted tractography The SM was reconstructed using high angle, small step-size tracts to capture the particularly small and highly angular path of the SM. The use of neuroanatomical expertise during the planning, cleanup and checking processes strengthened the face validity of the technique. Reconstructing the SM in a reliable and accurate fashion may contribute to research into neuropsychiatric disorders and treatments.

## Author Contributions

DWR: recruitment, protocol design, data analysis, manuscript preparation and neuroanatomy. ER: recruitment, protocol design, tractograpy, data analysis and manuscript preparation. SR and SA: recruitment and tractography. CF, KD and LT: recruitment. KJL: data analysis and neuroanatomy. DB and PT: protocol design and neuroanatomy. TF: principal investigator. VO: principal investigator and manuscript preparation. EO: recruitment, protocol design, data analysis and manuscript preparation.

## Conflict of Interest Statement

The authors declare that the research was conducted in the absence of any commercial or financial relationships that could be construed as a potential conflict of interest.

## Footnotes

^1^http://www.exploredti.com

^2^https://www.mathworks.com/products/matlab.html

^3^http://surfer.nmr.mgh.harvard.edu/

^4^https://www.ibm.com/analytics/us/en/technology/spss/
